# Quality of diabetes care in Dutch care groups: no differences between diabetes patients with and without co-morbidity

**DOI:** 10.5334/ijic.1141

**Published:** 2013-12-23

**Authors:** Simone R. de Bruin, Sandra H. van Oostrom, Hanneke W. Drewes, Janneke T. de Jong-van Til, Caroline A. Baan, Jeroen N. Struijs

**Affiliations:** National Institute for Public Health and the Environment, Centre for Nutrition, Prevention and Health Services, The Netherlands; National Institute for Public Health and the Environment, Centre for Nutrition, Prevention and Health Services, The Netherlands; National Institute for Public Health and the Environment, Centre for Nutrition, Prevention and Health Services, The Netherlands; National Institute for Public Health and the Environment, Centre for Nutrition, Prevention and Health Services, The Netherlands; National Institute for Public Health and the Environment, Centre for Nutrition, Prevention and Health Services, The Netherlands; National Institute for Public Health and the Environment, Centre for Nutrition, Prevention and Health Services, The Netherlands

**Keywords:** bundled payment, care groups, co-morbidity, diabetes, disease management, integrated care, quality of care

## Abstract

**Objective:**

To evaluate the relationship between presence and nature of co-morbidity and quality of care for diabetes patients enrolled in diabetes disease management programmes provided by care groups.

**Methods:**

We performed an observational study within eight Dutch diabetes care groups. Data from patient record systems of care groups and patient questionnaires were used to determine quality of care. Quality of care was measured as provision of the recommended diabetes care, patients’ achievement of recommended clinical outcomes and patients’ perception of coordination and integration of care.

**Results:**

527 diabetes patients without and 1187 diabetes patients with co-morbidity were included. Of the co-morbid patients, 7.8% had concordant co-morbid conditions only, 63.8% had discordant co-morbid diseases only and 28.4% had both types of conditions. Hardly any differences were observed between patients with and without co-morbidity in terms of provided care, achievement of clinical outcomes and perceived coordination and integration of care.

**Conclusions:**

Our study implies that care groups are able to provide similar quality of diabetes care for diabetes patients with and without co-morbidity. Considering the expected developments regarding additional disease management programmes in care groups, it is of importance to monitor quality of care, including patient experiences, for all chronic diseases. It will then become clear whether accountable provider-led organisations such as care groups are able to ensure quality of care for the increasing number of patients with multiple chronic conditions.

## Introduction

In the Netherlands, like in other Western countries, numerous initiatives have been undertaken to enhance effectiveness and quality of diabetes management, often involving multidisciplinary cooperation in disease management programmes. In broad terms, disease management refers to a patient-centred approach that aims to structure and coordinate delivery of health care services to a specific patient group. Core elements of disease management programmes are: (1) a well-coordinated and proactive approach to health care needs; (2) patient-centredness by involving older people in decision-making and planning their care process, and by taking their individual needs into account; (3) (simultaneous) delivery of multiple interventions; and (4) involvement of professionals from multiple disciplines [1,2].

To expedite the implementation of disease management programmes, the Dutch Ministry of Health, Welfare and Sport introduced a new payment mechanism for disease management programmes, known as bundled payment [3,4]. This approach enables health insurers to purchase the different components of disease management programmes as a single package from care groups. Care groups are groups of associated care providers, often exclusively general practitioners, who are responsible for coordinating and ensuring the delivery of services included in the disease management programme [4,5]. In some aspects, the Dutch bundled payment model is comparable to payment reforms that are being introduced in the United States (i.e. accountable care organisations) and England (i.e. clinical commissioning groups) [6–8]. All concepts, for instance, aim to establish financial alignment of care providers by introducing new provider-led entities in the health care system that becomes financially and clinically accountable for the population they serve [4].

Although recent studies suggest that between 44% and 73% of the diabetic population is co-morbid [9,10], co-morbidity is not specifically addressed in Dutch diabetes disease management programmes provided by the care groups. Diabetes disease management programmes are delivered in conformity with the Dutch Diabetes Federation Health Care Standard ***, which is limited to the requirements for generic diabetes care [11]. Hence, (additional) requirements for care services for co-morbid patients are not defined.

Diabetes control in patients with co-morbidities is, however, challenging due to multiple and possibly competing treatment demands [12–17]. Moreover, patients with multiple chronic conditions usually require the involvement of a large number of health care providers. Coordination of care is therefore essential, yet alignment between the different health care providers often fails. As a result, patients with multiple chronic conditions are prone to receive fragmented, incomplete, inefficient and ineffective care [15,18–20].

During our study, in most care groups treatment for other chronic illnesses continued to be offered through usual primary care. This implies that, just as diabetes patients without co-morbidity, also diabetes patients with co-morbidity mostly participated in only one disease management programme. Care groups did not offer (integrated) interventions specifically targeted at patients with co-morbidity [21]. Up until now, it is unknown whether care groups are able to comply with the Dutch Diabetes Federation Health Care Standard for diabetes patients with co-morbidity and whether co-morbid patients are able to achieve clinical outcomes formulated in the Dutch Diabetes Federation Health Care Standard. Moreover, it is unknown whether the single-disease approach in disease management programme has consequences for the coordination and integration of care for diabetes patients with co-morbidity. These insights are, however, important since care groups assume clinical and financial responsibility for all assigned diabetes patients, including those with co-morbidity. Consequently, they are obliged to report accountability information to health insurers that provides insight into the quality of diabetes care delivered. Based on their performance, care groups might receive either additional or reduced payments [4,5].

The present study therefore evaluated quality of diabetes care for diabetes patients with and without co-morbidity enrolled in diabetes disease management programmes provided by care groups. Since it is assumed that quality of care may differ across co-morbidity types [15], we additionally evaluated whether quality of care for diabetes patients with co-morbidity was related to the nature of their co-morbid diseases.

## Methods

### Study design

This study was part of a larger evaluation study with three year follow-up including nine care groups involved in diabetes management. The study focused on the effects of bundled payment for diabetes care on the health care delivery process and quality of care provided [21]. Details about this evaluation study are reported elsewhere [4,5,21,22]. For the study described in the present paper, we used data from June 2009 to June 2010. Quality of care was measured by (1) provision of diabetes care recommended by the Dutch Diabetes Federation Health Care Standard, (2) patients’ achievement of recommended clinical outcomes recommended by the Dutch Diabetes Federation Health Care Standard and (3) patients’ perception of coordination and integration of care provided.

### Data collection

Data were retrieved from two data sources: (1) patient record systems of care groups and (2) patient questionnaires completed by patients enrolled in the disease management programmes of the care groups. Eight care groups delivered patient data from their patient record systems. In June 2010, we further administered patient questionnaires to a random sample of 4000 diabetes patients clustered within a random sample of 78 general practitioner practices representing these eight care groups. Patient questionnaires were additionally sent to a purposive sample of 377 patients who participated in an earlier study [4,5]. Patients were predominantly people with type 2 diabetes [23]. Data from patient questionnaires and the patient record systems were linked using identical, pseudonymous patient identification numbers. The response rate to the patient questionnaire was 46%, leading to a total number of 1714 diabetes patients whose data could be linked to the data from the patient record systems for the present study.

### Study variables

#### Provision of recommended care and patients’ achievement of recommended clinical outcomes

Provision of recommended care was operationalised based on process measures including yearly control of HbA1c level, low-density lipoprotein cholesterol, body mass index, systolic blood pressure (SBP), creatinine and examination of patient's feet and eyes. Patients’ achievement of recommended clinical outcomes was operationalised based on outcome measures including patients’ values on HbA1c level, low-density lipoprotein cholesterol, body mass index and systolic blood pressure. Most process and outcome measures were derived from the patient record systems of care groups to determine the overall percentages of patients who received diabetes care as recommended by the Dutch Diabetes Federation Health Care Standard and who reached recommended clinical outcomes, respectively, in the past year. Three process measures were derived from the patient questionnaire: patients’ receipt of dietary, exercise and smoking counselling as recommended by the Dutch Diabetes Federation Health Care Standard.

#### Patients’ perception of coordination and integration of diabetes care

For patients’ perception of coordination and integration of diabetes care, we used data from the patient questionnaire. Patients’ experiences with cooperation between health care providers and coordination of care were identified using six items of the questionnaire of the Dutch National Panel of the Chronically Ill and Disabled (NPCG). The items could be rated on a five-point response scale with higher scores referring to better coordination of care [24,25].

Patients’ experiences with integration of diabetes care were identified using the Patient Assessment of Chronic Illness Care [26]. The Patient Assessment of Chronic Illness Care consists of 20 items and measures the extent to which patients experience that diabetes care provided in the last 12 months was congruent with the chronic care model [27,28]. Also the items of the Patient Assessment of Chronic Illness Care could be rated on a five-point response scale with higher scores referring to a higher level of integration of diabetes care. An overall Patient Assessment of Chronic Illness Care score was computed by averaging scores on each of the items.

#### Co-morbid diseases

The presence of co-morbidities was determined through the patient questionnaire by self-report of patients. Patients were asked to indicate whether they suffered from a disease from one of the following 15 disease categories in the 12 months prior to the study: (1) retinopathy; (2) stroke or transient ischaemic attack; (3) heart attack; (4) other severe cardiovascular disease such as heart failure or angina pectoris; (5) cancer; (6) migraine headache or other type of regular headache; (7) atherosclerosis in abdomen or legs; (8) asthma, chronic bronchitis, emphysema or Chronic Obstructive Pulmonary Disease (COPD); (9) severe or persistent intestinal disorders (more than 3 months); (10) urinary incontinence; (11) severe or persistent back pain; (12) osteoarthritis; (13) rheumatoid arthritis; (14) other severe or persistent problems with neck or shoulder; and (15) other severe or persistent problems with elbow, wrist or hand. This list was derived from the Permanent Survey Living Conditions [29].

We categorised diabetes patients in two manners. First, we categorised patients into patients without and with co-morbidity. Second, we categorised patients in line with the theoretical framework of Piette and Kerr [15]. In line with this framework, we differentiated between different types of co-morbid diseases based on the degree to which their treatment was concordant with that for diabetes. Co-morbid diseases can be grouped into concordant diseases (i.e. diseases that overlap with diabetes in terms of pathogenesis and management plans [diseases (1–4) and (7)] and discordant diseases [i.e. diseases with unrelated pathogenesis or management plans; diseases (5, 6 and 8–15)]. We categorised diabetes patients into four categories: (1) patients without co-morbidity; (2) patients with concordant co-morbid diseases only; (3) patients with discordant co-morbid diseases only; and (4) patients with both concordant and discordant diseases.

#### Confounders

We included sex, age, education, ethnicity, diabetes type and diabetes duration as potential confounders. These data were derived from the patient record systems of the care groups and the patient questionnaire.

### Missing value imputation

Overall, the proportion of missing values per variable varied from 0% to 38.6%. We observed that baseline characteristics of patients with incomplete data differed from those with complete data. To prevent biased results, we imputed missing values since in such cases analyses based on multiple imputations are preferred over complete case analyses [30,31]. Missing values were imputed using Multivariate Imputation by Chained Equations (MICEs) procedure in R [32]. Statistical procedures used in Multivariate Imputation by Chained Equation are described by Van Buuren (2012) [33]. Twenty imputation datasets were created. Results of the regression analyses on the 20 imputed datasets were pooled by the MIANALYZE procedure in SAS.

### Data analysis

Descriptive analyses were applied to describe the characteristics of the total sample of diabetes patients with and without co-morbidity. Random effect models with two levels (level 1 patients and level 2 general practitioner practices) were used to compare quality of care between groups. Variation due to care groups was examined and found to be negligible. Therefore, care groups were not included as a level in the model. Process and clinical outcome indicators were binary variables, perceived coordination items were ordinal variables and the Patient Assessment of Chronic Illness Care score was a continuous variable. Generalised linear mixed models with binomial distribution and logit link function were used for the binary variables, ordered multinomial models with cumulative logit link for the ordinal variables [34] and generalised linear mixed models with Gaussian distribution and identity link function for the continuous variable. All analyses were adjusted for sex, age, education, ethnicity, diabetes type and diabetes duration. SAS (version 9.3) was used for the analyses and differences were considered significant at *p* ≤ 0.05.

## Results

### Patient characteristics

Table 1 presents the characteristics of our study population of which 30.7% (*n*=527) had no co-morbidities and 69.3% (*n*=1187) had at least one co-morbidity. Of the co-morbid patients, 32.4% (*n*=385) had one co-morbid condition, 24.5% (*n*=291) had two co-morbid conditions, 17.1% (*n*=203) had three co-morbid conditions and 26.0% (*n*=308) had four or more co-morbid conditions (data not shown). The average number of co-morbidities was 2.8. Of the co-morbid patients, 7.8% had concordant co-morbid conditions only, 63.8% had discordant co-morbid diseases only and 28.4% had both types of conditions. Overall, musculoskeletal diseases were most prevalent. The mean age of diabetes patients with co-morbidity was higher than that of diabetes patients without co-morbidity (67.8 years vs. 64.6 years) and a larger proportion of patients were women (54.1% vs. 39.5%). Although diabetes disease management programmes in principally focus on patients with type 2 diabetes, some care groups also include patients with type 1 diabetes in the disease management programme. In our study population, 3.2% of people without co-morbidity and 1.8% with co-morbidity were type 1 diabetes patients. Annex 1 presents the characteristics of diabetes patients per co-morbidity type.

### Provision of recommended diabetes care

No differences were observed between diabetes patients without and with co-morbidity with regard to proportions of patients who received diabetes care as recommended by the Dutch Diabetes Federation Health Care Standard (Table 2). For almost none of the process measures, a relationship was found between the nature of co-morbid diseases and the likelihood to receive recommended diabetes care. Only HbA1c control was significantly less likely in patients with both concordant and discordant co-morbid diseases (OR, 0.47; 95% CI, 0.26–0.84) than in patients without co-morbidity.

### Patients’ achievement of recommended clinical outcomes

With the exception of the proportion of patients with a body mass index lower than 25 kg/m^2^, no differences were observed in the proportions of patients who reached clinical outcomes between diabetes patients with or without co-morbidity (Table 2). Patients with co-morbidity were significantly less likely than their counterparts without co-morbidity to have a body mass index lower than 25 kg/m^2^ (OR, 0.70; 95% CI, 0.51–0.96). After categorising patients by the nature of their co-morbid diseases, we observed that only the difference in body mass index between patients with discordant co-morbid diseases only and patients without co-morbid diseases was still significant.

### Patients’ perception of coordination and integration of diabetes care

Three aspects related to coordination of care (i.e. whether health care providers made good arrangements with one another, whether health care providers were well-informed and whether patients had to repeat their stories) were rated significantly lower by patients with co-morbidity than by patients without co-morbidity (Table 2). After categorising patients by the nature of their co-morbid diseases, we observed that two aspects of coordination of care (i.e. whether health care providers were well-informed and whether patients had to repeat their stories) were rated significantly lower by patients with discordant diseases only than by patients without co-morbidity. The aspect ‘whether patients had to repeat their stories’ was also significantly lower rated by patients with concordant as well as discordant diseases than by patients without co-morbidity. Annex 2 presents per aspect of coordination of care the frequency of responses per category (1–5). Experiences of patients with integration of diabetes care did not differ significantly between patients with and without co-morbidity (Table 2). No relationship was observed between the nature of co-morbid diseases and experienced integration of diabetes care either.

## Discussion

### Interpretation of findings

This study evaluated quality of care for diabetes patients with and without co-morbidity enrolled in diabetes disease management programmes provided by care groups. Our study revealed hardly any differences in quality of care for patients with and without co-morbidity. Hence, our study implies that the potentially competing treatment demands of patients with co-morbidity hardly affect quality of diabetes care provided by care groups.

Literature regarding the relationship between co-morbidity and quality of care is controversial. There is evidence that quality of care differs by co-morbidity type [35–37]. The study of Pentakota et al. (2012) [35], for example, suggests that discordant co-morbid conditions are associated with diminished diabetes care, whereas quality of diabetes care for diabetes patients with concordant co-morbid conditions is similar compared to diabetes patients without co-morbidity. Also the study of Urrutia et al. (2012) [37] confirms that discordant co-morbid conditions (i.e. anxiety and depression in this study) adversely affect quality of care. However, findings of other studies are more in line with our findings and suggest that quality of care for patients with co-morbidity is similar to or even better than for patients without co-morbidity [16,38–40].

A possible explanation for hardly finding any differences in quality of care between diabetes patients with and without co-morbidity in our study may be related to the expertise of health care providers involved in diabetes care. Health care providers involved in care groups who were interviewed for a study related to the current study indicated to have always been accustomed to responding to patients’ comprehensive care needs. General practitioners for example stated that the generalist nature of their discipline makes them highly suited to provide integrated care and that quality of diabetes care is therefore not necessarily worse for patients with co-morbidity than for patients without co-morbidity. According to them, the single-disease approach in the bundled payment system and in the disease management programme does not interfere with how diabetes care is being delivered. They therefore indicated not to experience any major problems in relation to the care for diabetes patients with co-morbidity [21]. This may also be reflected by recent figures on diabetes patients in primary care. Although 70% of diabetes patients in primary care has co-morbid conditions of which about 60% has at least two co-morbid conditions [10], still the large majority only utilises diabetes care in the primary care setting. Only 10% of diabetes type 2 patients utilies specialist care in the hospital settings. It is likely that particularly more complex cases (e.g. those with severe complications) are referred to specialist care [41]. This implies that all other diabetes patients, despite their co-morbid conditions, are treated in primary care and included in a disease management programme.

Another explanation for our findings may be that most care groups were still contracting only one or two disease management programmes. Treatment for other illnesses continued to be offered through usual primary care and reimbursed via the old pricing system (e.g. fee-for-service for most care providers). Compliance with care as recommended by the disease management programme and achievement of recommended clinical outcomes may therefore have been manageable for the majority of patients. This may also explain why patients with and without co-morbidity experienced fairly similar coordination and integration of diabetes care.

Although quality of care was overall similar for diabetes patients with and without co-morbidity, some exceptions should be noted. Compared to diabetes patients without co-morbidity, HbA1c control was significantly less likely in patients with both concordant and discordant co-morbid diseases. The difference was, however, small and still the large majority (>95%) of patients received the recommended care. Another exception was the finding that patients with co-morbidity were significantly less likely to reach the recommended clinical outcome for body mass index than patients without co-morbidity. Compared to the achievement of the other treatment goals, the proportion of patients who were able to reach the treatment goal for body mass index was low. It is, however, questionable whether this finding is the consequence of diminished quality of care to co-morbid diabetes patients. A more likely explanation is the relationship between overweight and obesity and having multiple chronic conditions [42].

Also, the difference in perceived coordination of diabetes care was an exception. Patients with co-morbidity, particularly those with discordant co-morbid diseases only and discordant as well as concordant diseases, seemed to perceive less coordination of diabetes care. It should, however, be noted that differences between patients from the different groups were small, and that the majority of patients from the different groups experienced good coordination of diabetes care.

### Methodological considerations

In previous studies, quality of care was evaluated by clinical process and/or outcome measures only [16,35,38]. However, also coordination and integration of care are increasingly being recognised as important indicators for quality of care [20,43,44]. A strength of our study was that we addressed multiple domains of quality of care. To measure perceived integration of diabetes care, we used the Patient Assessment of Chronic Illness Care, which is according to the literature one of the most promising instruments to measure patients’ experiences with integration of chronic care [45]. A recent study suggests, however, that some of the Patient Assessment of Chronic Illness Care items are not applicable for all patients [22]. Improvement of the Patient Assessment of Chronic Illness Care is therefore necessary to increase its usefulness and our findings should therefore be interpreted with some caution.

Our study did not incorporate any data on disease severity of diabetes patients. It was therefore not possible to determine and compare the health status of diabetes patients with and without co-morbidity. The number and types of co-morbid conditions thus served as a proxy for the health status of the diabetes patients who participated in this study. Since, as stated before, the large majority of diabetes patients is being treated in primary care [21], we assumed that there was sufficient variation in health status across diabetes patients to potentially detect differences in quality of care.

Our study was performed among eight Dutch care groups. Currently, there are about 100 care groups in the Netherlands consisting of an average of 78 general practitioners who deliver care to an average of 6455 diabetes patients each [21]. Characteristics of the eight care groups participating in the present study, in terms of e.g. the average number of general practitioners, inclusion of patients with co-morbidity and number of implemented or planned additional disease management programmes, were reasonably similar to those of 55 care groups involved in another recent study [46]. This suggests that our findings are representative for other Dutch care groups. We further compared characteristics of respondents to the questionnaire with those of the total population of diabetes patients enrolled in disease management programmes of the eight care groups. No substantial differences in terms of e.g. gender, age and diabetes duration were identified between them [21]. Our study population may therefore be considered as a representative sample of care groups’ patients.

An existing list was used to identify co-morbid diseases in the participating diabetes patients. This list is composed of diseases that are prevalent in at least 1% of the Dutch population, which are sufficiently severe and understandable for respondents. The consequence of using a list based on these principles is that other prevalent diseases in diabetes patients, such as depression, are not taken into account. Moreover, diseases were self-reported. It is known that the method used to identify co-morbid diseases influences the prevalence figures [47]. Hence, we may not have gained insight into the full spectrum of chronic diseases in our study population. Moreover, this method provided no insight into the severity of each of the co-morbid diseases.

### Recommendations

Since many care groups are planning additional disease management programmes for other chronic diseases (i.e. COPD, vascular risk management and dementia) or have already implemented them, monitoring of quality of care within and outside each of the programs, including patient experiences, remains necessary to ensure quality and continuity of care in care groups. These developments may result into competing interests of care groups and patients with multiple chronic diseases. Due to their accountability for diabetes care, delivery of recommended care and achievement of recommended clinical outcomes serve the interests of care groups. Rigid adherence to recommended care for patients with multiple chronic conditions, however, may conflict with the main principle of disease management programmes, i.e. patient-centredness. Particularly since patients with multiple chronic conditions will be included in multiple disease management programmes and thus have to deal with multiple health care providers who all aim to deliver recommended care. Within this context, the development of quality indicators, which align the patient-centeredness principle of disease management programmes and the accountability obligations of care groups, needs to be considered. This may also increase insight into for which patients quality of care can be maintained by participation in different disease-specific programmes and for which patients a more generic approach (e.g. through case management) is required.

## Conclusion

Our study implies that care groups are able to provide similar quality of diabetes care for diabetes patients with and without co-morbidity enrolled in a diabetes disease management programme. In view of the expected developments regarding additional disease management programmes in care groups, it is of importance to monitor quality of care, including patient experiences, for all chronic diseases. It will then become clear whether accountable provider-led organisations such as care groups are able to ensure quality of care for the increasing number of patients with multiple chronic conditions.

## Figures and Tables

**Table 1.**
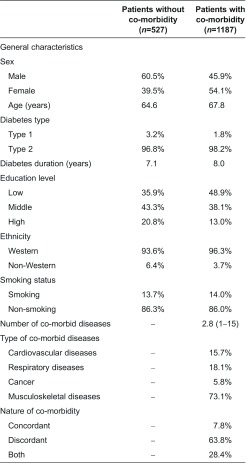
Characteristics of diabetes patients with and without co-morbidity. Data shown as mean or as frequency (%)^a^

^a^Means and average frequency over the 20 imputed datasets.

**Table 2.**
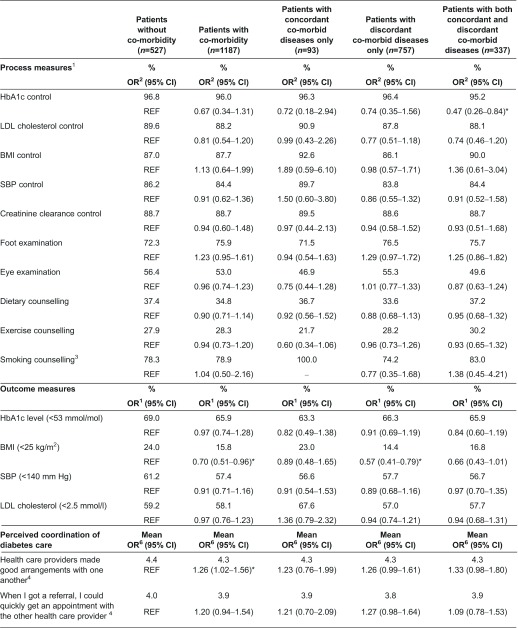

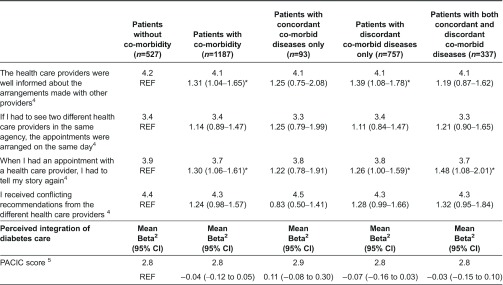
Proportions of patients who received recommended diabetes care and who achieved recommended clinical outcomes in the year prior to the study and patients’ rating of coordination and integration of diabetes care

^1^Controlled once in the previous year.

^2^Adjusted for sex, age, education, ethnicity, diabetes type and diabetes duration.

^3^Smoking counselling was only reported by smokers: *n*=72 diabetes patients without co-morbidity, *n*=131 diabetes patients with co-morbidity, *n*=14 diabetes patients with concordant co-morbid diseases only, *n*=105 diabetes patients with discordant co-morbid diseases only and *n*=47 diabetes patients with concordant and discordant co-morbid diseases. All patients with concordant co-morbid diseases only received smoking counselling; therefore, no odds ratio could be calculated.

^4^Scores range from 1 to 5, higher scores refer to better coordination of diabetes care.

^5^Scores range from 1 to 5, higher scores refer to a higher level of integration of diabetes care.

^6^OR<1 refers to better coordination of care (using ordered multinomial regression).

*p ≤ 0.05.

**Annex 1.**
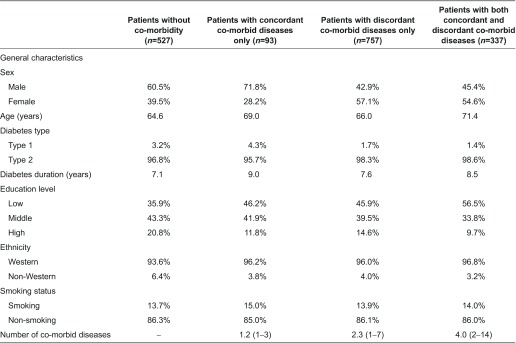
Characteristics of diabetes patients without co-morbidity and with different types of co-morbidity. Data shown as mean or as frequency (%)^a^

^a^Means and average frequency over the 20 imputed datasets.

**Annex 2.**
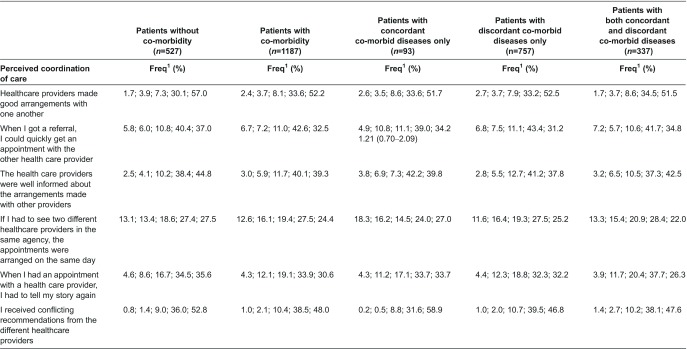
Perceived coordination of diabetes care by patients without and with different types of co-morbid diseases (%)

^1^Frequency of responses in category 1–5 of the Dutch National Panel of the Chronically Ill and Disabled (NPCG). Scores range from 1 to 5, higher scores refer to better coordination of diabetes care.
